# Conversion of Racemic Alkyl Aryl Sulfoxides into Pure Enantiomers Using a Recycle Photoreactor: Tandem Use of Chromatography on Chiral Support and Photoracemization on Solid Support

**DOI:** 10.1021/acs.joc.3c00265

**Published:** 2023-05-08

**Authors:** Kumi Tozawa, Kosho Makino, Yuki Tanaka, Kayo Nakamura, Akiko Inagaki, Hidetsugu Tabata, Tetsuta Oshitari, Hideaki Natsugari, Noritaka Kuroda, Kunio Kanemaru, Yuji Oda, Hideyo Takahashi

**Affiliations:** † Faculty of Pharmaceutical Sciences, Tokyo University of Science, 2641 Yamazaki, Noda-shi, Chiba 278-8510, Japan; ‡ Research Institute of Pharmaceutical Sciences, Musashino University, Nishitokyo, Tokyo 202-8585, Japan; § Faculty of Science and Technology, Seikei University, 3-3-1 Kichijoji Kitamachi, Musashino-shi, Tokyo 180-8633, Japan; ∥ Faculty of Pharma Sciences, Teikyo University, 2-11-1 Kaga, Itabashi-ku, Tokyo 173-8605, Japan; ⊥ Graduate School of Pharmaceutical Science, The University of Tokyo, 7-3-1 Hongo, Bunkyo-ku, Tokyo 113-0033, Japan; # YMC Co., Ltd., 284 Daigo, Karasuma Nishiiru Gojo-dori, Shimogyo-ku, Kyoto 600-8106, Japan; ¶ IWASAKI ELECTRIC CO., LTD., 1-1, Ichiriyama-cho, Gyoda-shi, Saitama 361-8505, Japan

## Abstract

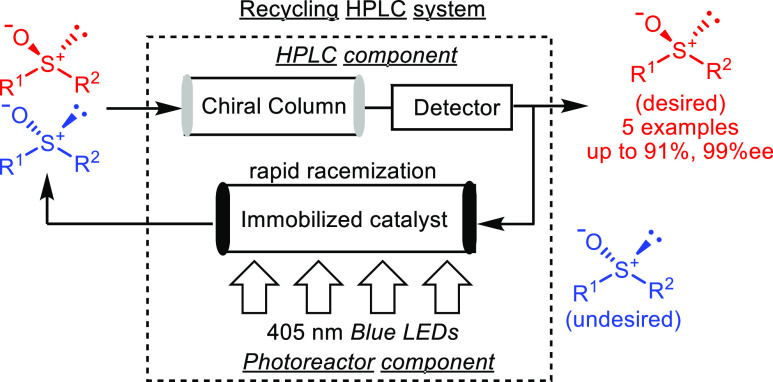

Chiral sulfoxides are valuable in the fields of medicinal chemistry and organic synthesis. A recycle photoreactor utilizing the concept of deracemization, where a racemate is converted into a pure enantiomer, is developed and successfully applied in the syntheses of chiral alkyl aryl sulfoxides. The recycling system consists of rapid photoracemization using an immobilized photosensitizer and separation of the enantiomers via chiral high-performance liquid chromatography, and the desired pure chiral sulfoxides are obtained after 4–6 cycles. The key to the success of the system is the photoreactor site, wherein the photosensitizer 2,4,6-triphenylpyrylium is immobilized on the resin and irradiated (405 nm) to enable the rapid photoracemizations of the sulfoxides. As the green recycle photoreactor requires no chiral components, it should be a useful alternative system for application in producing chiral compounds.

## Introduction

**Figure 1:**
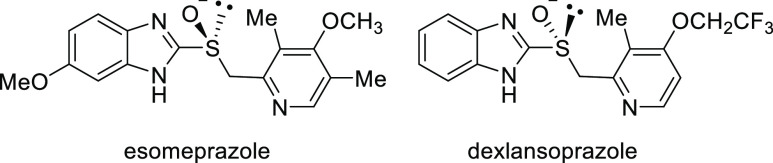
Antiulcer drugs containing chiral sulfoxides.

Recycling high-performance liquid chromatography (HPLC) enables the recycling of samples to increase the efficiency of separation. It is widely used to isolate and purify enantiomers, diastereomers, and various structurally related or unrelated compounds.^1a−1e^ Given its usefulness, incorporating additional devices into the recycling system to enhance its functionality should attract increased attention. From this perspective, we focused on the conversion of a racemate to a single enantiomer using chiral HPLC,^2a−2f^ which may be summarized by the following process: (1) isolate each enantiomer, (2) racemize the undesired enantiomer, and (3) recycle the racemic mixture by subjecting it to the resolution conditions. Using this approach, only five cycles are theoretically required to produce the enantiomerically pure compound in a yield of >96%. Much effort has been focused on the enantiomeric enrichment of stereolabile chiral compounds using HPLC methods. However, even if on-column stopped-flow bidimensional recycling HPLC is employed,^2d^ the procedure is so complicated that the potential of the deracemization-recycling approach has not been realized. A key solution to this problem may be the use of an on-column rapid photoracemization reaction. Therefore, we developed a recycling HPLC system equipped with an immobilized photoracemization site to realize a rapid reaction, which should represent a powerful tool in generating a pure enantiomer. Compared to thermal reactions, photoreactions^3a−3d^ are more practical in terms of green, sustainable chemistry. In this regard, Kappe et al. reported efficient photochemical deracemization using a flow system.^3e^ In contrast, rapid, reversible photoracemization without heating was integrated with the flow system of the recycling HPLC. Among numerous deracemization approaches,^4a−4l^ the photoracemizations of chiral sulfoxides^5a−5e^ are the most promising reactions for application in our system. Chiral sulfoxides are critical bioactive compounds and intermediates in chemical reactions.^6a−6e^ Chiral sulfoxides, e.g., esomeprazole^7a,7b^ and dexlansoprazole,^7a,7c^ also attract attention as antiulcer drugs (Figure 1).

The characteristic properties of chiral sulfoxides include their thermal stabilities. The pyramidal inversion of the S center requires 159.1–171.7 kJ/mol, which entails extreme temperatures (approximately 200 °C).^8a,8b^ In contrast, the racemizations of chiral sulfoxides are facilitated by light irradiation.^9a,9b^

**Scheme 1:**
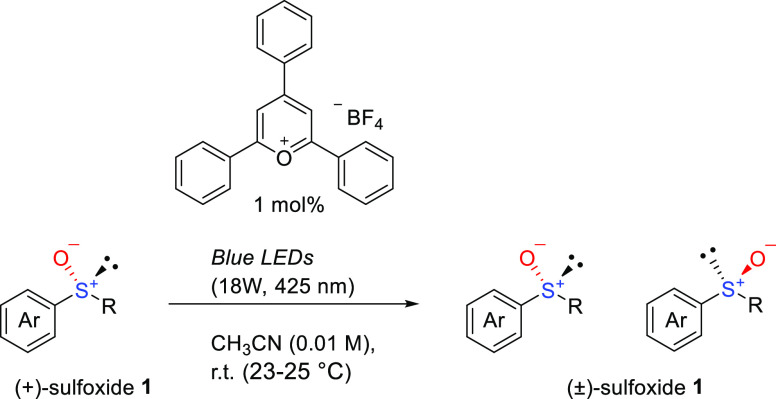
Rapid Photoracemization in the Presence of TPT^+^

Since the initial reports by Mislow,^9a^ the pyramidal inversions of alkyl aryl sulfoxides in the presence of photosensitizers have been investigated,^10^ and S inversion may occur in an exciplex comprising the photosensitizer and sulfoxide.^11a,11b^ Recently, we reported that the use of 1 mol % of the photosensitizer 2,4,6-triphenylpyrylium tetrafluoroborate (TPT^+^) enables the rapid photoracemizations of chiral alkyl aryl sulfoxides **1**. Under irradiation, no photoracemization occurred without a photocatalyst. Various substitution patterns are tolerated, and the racemizations proceed extremely rapidly (*k*_2_ = 1.77 × 10^4^–6.08 × 10^1^ M^–1^ s^–1^, *t*_1/2_ = 0.4–114 s, Scheme 1).^12^

The rapid photoracemizations of chiral sulfoxides should be applicable in deracemization. Although the one-pot deracemization of chiral sulfoxides using chemoenzymatic methods has been reported,^13^ to the best of our knowledge, the deracemization of chiral sulfoxides utilizing an on-column rapid photoracemization reaction has never been reported. We herein report the rapid photoracemizations of chiral sulfoxides on the solid phase, which is incorporated into a recycling HPLC system. This system, denoted the recycle photoreactor, consists of two steps: (1) isolation of the desired enantiomer and (2) rapid racemization of the other enantiomer to produce an enantiomerically pure sulfoxide in a good yield. The rapid photoracemization on the solid phase is critical.

## Results and Discussion

### Preparation of Immobilized Photosensitizer

**Scheme 2:**
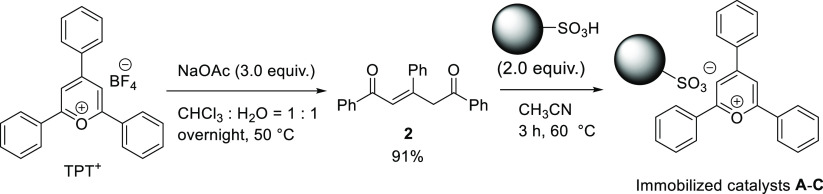
Preparation of Immobilized Catalysts

To realize a rapid photoracemization in the photoreactor within the recycling HPLC system, the photosensitizer TPT^+^ should be immobilized on the solid phase. We analyzed commercially available supporting materials, and the cation-exchange resin was the most suitable because cationic TPT^+^ could be ionically bonded to the sulfonic acid anion (RSO_3_^–^) of the resin.^14^ Initially, TPT^+^ was immobilized on commercial cation-exchange resin via ion exchange in H_2_O. However, the removal of BF_4_^–^ from the prepared immobilized catalysts was challenging, and multiple cumbersome post-processing steps, such as washing with various solvents, were necessary. Furthermore, the BF_4_^–^ contaminant hindered the estimation of the loading ratio of TPT^+^, and thus, an alternative procedure was developed. 1,3,5-Triphenyl-2-pentene-1,5-dione (**2**),^15^ which is prepared using TPT^+^ via hydrolysis with sodium acetate (NaOAc), is treated with several cation-exchange resins containing sulfonic acid to generate ionically bonded immobilized catalysts without counter ions (Scheme 2).

Using this procedure, we may calculate the loading ratio of **2** based on the recovered amount of free **2** (Table 1), and immobilized catalysts **A**–**C** attached to the resin exhibit good loading ratios. To evaluate the catalytic properties of **A**–**C**, the photoracemization of enantiopure methyl *p*-tolyl sulfoxide {(+)-**1a**, >98% ee} in CH_3_CN served as a model reaction (Table 1). We screened a series of chiral columns, and CHIRAL ART Amylose-SA with CH_3_CN as the mobile phase may separate the racemate **1a**(12) into the individual enantiomers. To evaluate the racemization activities (*k*_obs_) of **A**–**C**, we irradiated (425 nm) a 10 mM solution of (+)-**1a** in the presence of 5 mg of each immobilized catalyst (Table 1 and Supporting Information Figures S1–S3). After the racemization was completed, the solid catalyst was removed by filtration. The filtrate was evaporated, and the residue was analyzed using HPLC and ^1^H nuclear magnetic resonance (NMR) spectroscopy to confirm that no other products were formed.

**Table 1:**
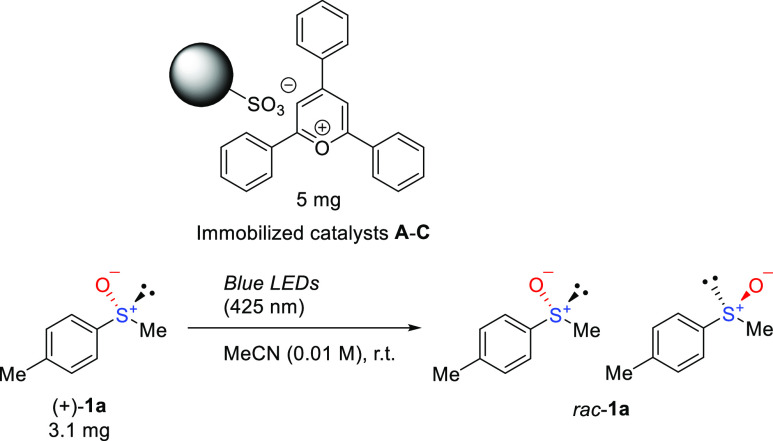
Evaluation of Immobilized Catalysts

entry	immobilized catalyst	resin	catalyst loading (mmol/g)	*k*_obs_ (s^–1^)	leaching
1	**A**	Dowex 50WX2 200–400 mesh	0.973	2.59 × 10^–2^	positive
2	**B**	Dowex 50WX4 200–400 mesh	0.980	9.73 × 10^–3^	positive
3	**C**	Dowex 50WX8 200–400 mesh	0.976	1.99 × 10^–2^	negative

Although **B** displays a somewhat low activity, **A** (*k*_obs_ = 2.59 × 10^–2^ M^–1^ s^–1^) and **C** (*k*_obs_ = 1.99 × 10^–2^ M^–1^ s^–1^) yield desirable results. Immobilized TP^+^ exhibits a comparable catalytic activity^12^ to that of soluble TPT^+^.

The possibilities of the leaching of TP^+^ from **A**–**C** were then examined following the model reaction. After irradiation (425 nm) for 2 min, the solid catalyst was separated by filtration, and the possibility of photoracemization in the filtrate due to catalyst leaching was examined. Photoracemizations caused by catalyst leaching are observed in the filtrates of **A** and **B**. However, no photoracemization is observed in the filtrate of **C**, which indicates that no leaching of TP^+^ into the solvent occurs (see Supporting Information, Figures S4–S7). Given the good results obtained using **C**, we then explored catalyst recyclability, and recycling studies of **C** were conducted using the model reaction. The reaction conditions were the same as those described above, and the progress of each run was determined by the enantiomeric excess of the recovered **1a**. After completion of each run (10 min), the solid catalyst was separated by filtration, washed successively with CH_3_CN and CH_2_Cl_2_, dried, and then reused in the subsequent run. **C** was reused up to 10 times, with the reaction rate remaining almost unchanged (Supporting Information, Table S1). Considering that Dowex 50WX8 produced the best results, the highest cross-linkage (8%) of styrene divinylbenzene in Dowex 50WX8 might be the key to tightly bond TP^+^ and resin.

### Development of the Recycle Photoreactor

**Figure 2:**
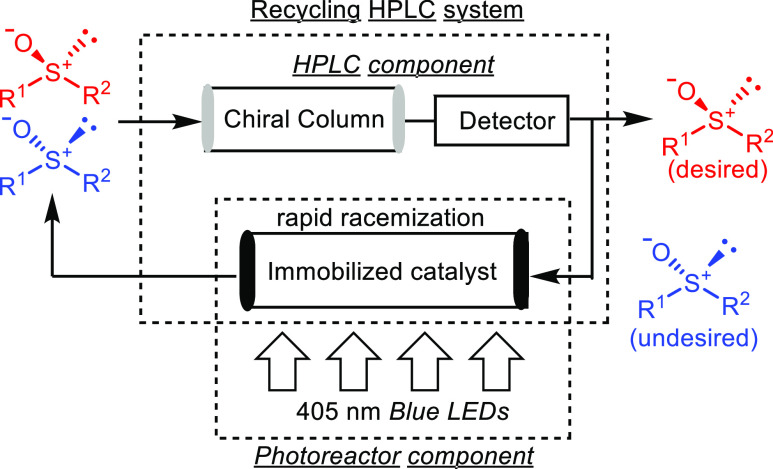
General flowchart representing the recycle photoreactor.

After preparing a suitable immobilized catalyst, we proceeded to construct the recycle photoreactor. This system comprises a photoreactor to enable rapid racemization and a chiral HPLC component to separate each enantiomer. For the photoreactor, **C** is packed into a glass tube (ϕ: 5 mm, length: 21 cm), which is covered with a device that irradiates light. In this device, a light-emitting diode (LED) tape (λ = 405 nm) is attached to an alumina block that maintains the constant temperature of the photoreaction. The photoreactor incorporated into the recycling HPLC system should racemize the chiral sulfoxide rapidly under light irradiation (Figure 2). To realize deracemization in this system, rapid photoracemization on the solid phase should be conducted under continuous flow conditions.

Initially, the glass tube containing **C** is inserted into the device and irradiated under LED light (405 nm). However, **C** adjacent to the inner wall of the glass tube darkens in color due to irradiation. The light absorption of TP^+^ immobilized on the resin adjacent to the inner wall may be too intensive, leading to self-destructive reactions, whereas most **C** in the central region of the glass tube remains under poor lighting conditions. Increasing light transmission to the central region of the glass tube should be key to success, and thus, light-permeable glass beads, which are almost identical in radius to **C**, are employed to yield a more efficient light distribution. A total of 6 g of a mixture of the glass beads and **C** is uniformly mixed and packed into the glass tube, and the efficiencies of the photoreactions with different ratios of glass beads and **C** were evaluated via the photoracemization of 2-methoxyethyl phenyl sulfoxide (+)-**1b**.^12^ The injected (+)-**1b** flows at 2.0 mL/min through the packed glass tube under optical irradiation (405 nm). The progress of the reaction was evaluated via in-line HPLC using a chiral column (CHIRALPAK ID).

The reaction rate increases as the proportion of glass beads increases, indicating the significance of light permeability in the photoreactor. However, using >3 wt % **C** leads to self-destructive reactions, and the absorption of more light by the resin adjacent to the inner wall of the glass tube compared to that absorbed by the central region appears inevitable.

In contrast, using <2 wt % **C** decreases the efficiency of racemization (Table 2, entries 2 and 3), and thus, using approximately 2 wt % **C** is optimal to enable sufficient racemization (Table 2, entry 1). In addition to (+)-**1b**,^12^ cyclopropyl phenyl sulfoxide (+)-**1c**^12^ and methoxyphenyl methyl sulfoxide (+)-**1d**^12^ were examined, and rapid racemizations are observed in both cases (Table 2, entries 4 and 5 and Supporting Information, Figures S8–S12). Although complete racemizations are not observed in the first runs (Table 2, entries 1, 4, and 5), successive cycles should increase the overall degrees of deracemization. After determining the optimized conditions for the photoreactor, we investigated the deracemization-recycling approach using the complete recycling HPLC system.

**Table 2:**
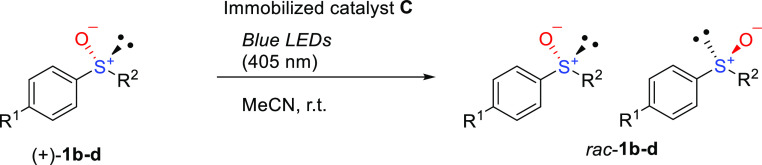
Racemization in the Flow Process

entry		R^1^	R^2^	ratio of **C**^a^ (% w/w)	optical purity (% ee)
1	**1b**	H	CH_2_CH_2_OMe	2.1	6
2	**1b**	H	CH_2_CH_2_OMe	1.7	24
3	**1b**	H	CH_2_CH_2_OMe	0.8	92
4	**1c**	H	cyclopropyl	2.1	4
5	**1d**	OMe	Me	2.1	6

a Percentage (% w/w) of immobilized catalyst **C** in a mixture of glass beads and immobilized catalyst **C** packed into a glass tube.

### Deracemizations of Alkyl Aryl Sulfoxides Using the Recycle Photoreactor

**Figure 3:**
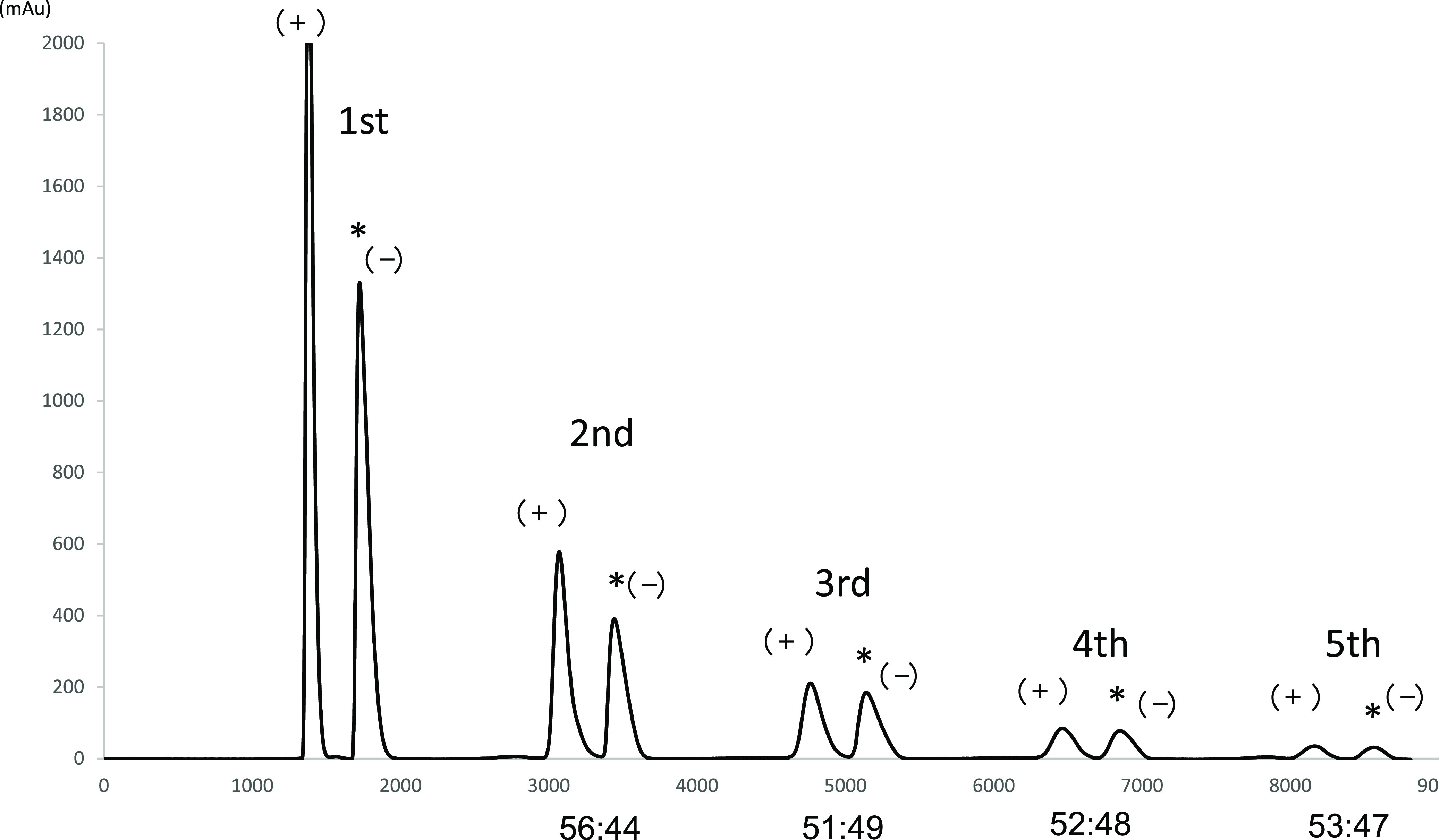
Enantiomeric enrichment of (−)-**1b** in the recycle photoreactor. Column: CHIRALPAK ID (ϕ: 10 mm, length: 50 cm), mobile phase: CH_3_CN, flow rate: 2.0 mL/min. The fractions of (−)-enantiomer, as denoted with asterisks, were collected and accumulated.

To generate chiral alkyl aryl sulfoxides, the following closed-loop recycling runs were conducted (Figure 3). Racemate **1b** (10 mg) was injected to the recycling HPLC equipped with the photoreactor and separated by the HPLC component (CHIRALPAK ID with CH_3_CN as the mobile phase, Figure 3, 1st run). The desired (−)-**1b** fraction was collected and the undesired (+)-**1b** fraction was recycled and flowed at 2.0 mL/min through the photoreactor (ϕ: 5 mm, length: 21 cm) under optical irradiation (405 nm). During the passing of (+)-**1b** through the photoreactor, rapid photoracemization occurred and the isomerized **1b** was again separated via HPLC. The ratio of (+)-**1b**/(−)-**1b** is 56:44 (Figure 3, 2nd run), and (−)-**1b** was collected and (+)-**1b** was simultaneously applied in the next cycle. After the 3rd and 4th runs, the obtained ratios of (+)-**1b**/(−)-**1b** are 51:49 and 52:48, respectively.

Finally, after the 5th run, the ratio of (+)-**1b**/(−)-**1b** is 53:47. The desired (−)-**1b** is accumulated over five cycles in a yield of 91% with a 98% ee (Table 3, entry 1 and Figures S13–S14). Similarly, (−)-**1c** and (−)-**1d** were deracemized using the recycle photoreactor (see Supporting Information, Figures S15–S18). (−)-**1c** is accumulated over five cycles in a yield of 85% with a 98% ee (Table 3, entry 2). (−)-**1d** is accumulated over six cycles in a yield of 79% with a 99% ee (Table 3, entry 4). In these cases, (−)-**1b**, (−)-**1c**, and (−)-**1d**, which are eluted after their antipodes, are collected. (+)-**1c**, which is eluted before its antipode, is collected and accumulated over four cycles in a yield of 87% with a 99% ee (Table 3, entry 3 and Supporting Information Figures S19 and S20). The order of elution of the enantiomers did not affect the yield or enantiomeric purity (Table 3, entries 2 and 3).

**Table 3:**
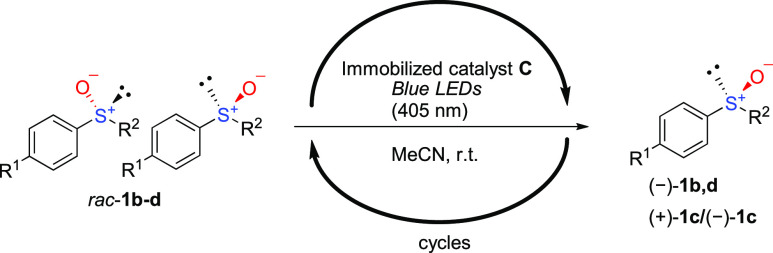
Scope of the Reaction^a^

entry	compound	R^1^	R^2^	number of cycles	yield (%)	optical purity (% ee)
1	(–)-**1b**	H	CH_2_CH_2_OMe	5	91	98
2	(–)-**1c**	H	cyclopropyl	5	85	98
3	(+)-**1c**	H	cyclopropyl	4	87	99
4	(–)-**1d**	OMe	Me	6	79	99

a HPLC conditions of recycle systems are described with their chromatograms in the Supporting Information.

Gradual broadening of the fraction peaks during later cycle runs complicated the separation process. Therefore, accumulated yields after several cycles were lower than expected. Careful selection of HPLC conditions is required, as the solvent used in HPLC is determined by the solvent used in photoracemization. In this case, CH_3_CN as the mobile phase was suitable for the resolution of enantiomers and photoracemization. These limitations should be addressed in future work. Nevertheless, a continuous deracemization process was accomplished using this recycling HPLC system.

## Conclusions

A recycle photoreactor was developed and applied in the resolution of chiral alkyl aryl sulfoxides **1**. As chiral sulfoxides occur within numerous bioactive compounds, developing an efficient method of producing the desired enantiomer should be very attractive in pharmaceutical applications. Notably, this green photoreaction system did not require any chiral components to provide enantiopure chiral sulfoxides.^7b,16^ Our procedure should also be applicable in the enantiospecific preparation of other racemic compounds. There are several issues to resolve, such as avoiding the gradual broadening of the fraction peaks during later cycle runs and preparing suitable separation conditions on the chiral column. If such problems are resolved, the scale-up and automatization of this system may yield a powerful tool to generate a pure enantiomer using racemic compounds without a cumbersome stereoselective synthesis or high-cost enantiopurification processes. Rapid photoracemization on the solid phase was critical to success, and improvements to the photoreactor system shall be reported in due course.

## Experimental Section

### General Information

All reagents were purchased from commercial suppliers and used as received. Reaction mixtures were stirred magnetically, and the reactions were monitored using thin-layer chromatography on precoated silica gel plates. An oil bath was used for the reactions that require heating. Column chromatography was performed using silica gel (45–60 μm) and extracted solutions were dried over anhydrous Na_2_SO_4_. Solvents were evaporated under reduced pressure, and ^1^H and ^13^C NMR spectroscopy was performed at 296 K at 600 and 150 MHz, respectively, unless otherwise stated. Tetramethylsilane (TMS, δ 0.00) or residual internal CHCl_3_ (^1^H NMR: δ 7.26 and ^13^C NMR: δ 77.16) was used as an internal reference in ^1^H and ^13^C NMR spectroscopy of samples in CDCl_3_. Coupling constants (*J*) are reported in Hertz (Hz), and splitting patterns are abbreviated as follows: singlet (s); doublet (d); triplet (t); quartet (q); multiplet (m); and broad (br). High-resolution mass spectrometry was conducted using an electrospray ionization time-of-flight (TOF), atmospheric pressure chemical ionization-TOF, or electron impact mass spectrometer. Infrared (IR) spectroscopy was performed using a Fourier transform IR spectrometer equipped with attenuated total reflectance (diamond). Melting points (mps), which are uncorrected, were recorded using a melting point apparatus. Room temperature means 23–25 °C. An optical irradiation device (Evoluchem PhotoRedOx Box) and chemistry screening kits (HepatoChem Inc., MA, USA) were used for LED irradiation. In the photoreactor, the chiral sulfoxides were irradiated with a blue LED light tape (405 nm) (Figures S21 and S22).

### General Experimental Procedure

All resins were purchased from commercial suppliers and used as received, and the characterizations of **1a–d** are described in our previous study.^12^

### Synthesis of **2**(13)

NaOAc (1.24 g, 15.1 mmol) was added to a stirred solution of TPT^+^ (2.00 g, 5.05 mmol) in 25 mL of CHCl_3_:H_2_O (1:1 v/v). After stirring at 50 °C overnight, the mixture was poured into H_2_O and extracted using CH_2_Cl_2_. The resulting extract was washed with brine, dried, and concentrated. To remove counter ions, the residue was purified via column chromatography (silica gel, hexane/ethyl acetate = 4:1) to afford **2** (1.50 g, 4.60 mmol, 91%) as a colorless amorphous solid. mp: 114–116 °C.

### General Procedure for Preparing **A–C**

Dowex 50WX2 200–400 mesh (250 mg, Fujifilm Wako Pure Chemical, Osaka, Japan) was added to a stirred solution of **2** (81.6 mg, 0.25 mmol) in CH_3_CN (25 mL). After the mixture was stirred at 60 °C for 3 h, the solid catalyst was removed by filtration and stirred in CH_3_CN (200 mL) at room temperature for 3 h. After filtration, the solid catalyst was then stirred in CH_2_Cl_2_ (200 mL) at room temperature for 3 h. After filtration, the solid catalyst was dried in vacuo overnight to afford **A**. The washes were collected and evaporated to provide 2.2 mg of unreacted **2**, indicating that 97.3% of **2** was loaded on **A**. The catalyst loading was 0.973 mmol/g.

**B** was prepared according to the procedure described above for the preparation of **A**. **B** was prepared using **2** (81.6 mg, 0.25 mmol) and Dowex 50WX4 200–400 mesh (250 mg, Fujifilm Wako Pure Chemical), and 1.6 mg of **2** was recovered, indicating that 98.1% of **2** was loaded on **B**. The catalyst loading was 0.980 mmol/g.

**C** was prepared according to the procedure described above for the preparation of **A**. **C** was prepared using **2** (81.6 mg, 0.25 mmol) and Dowex 50WX8 200–400 mesh (250 mg, Fujifilm Wako Pure Chemical), and 2.0 mg of **2** was recovered, indicating that 97.5% of **2** was loaded on **C**. The catalyst loading was 0.976 mmol/g.

### Evaluation of **A–C**

A solution containing (+)-**1a** (3.1 mg, 0.02 mmol) and **A**/**B**/**C** (5 mg) in CH_3_CN (2 mL) was stirred in a photoreactor equipped with blue LEDs (425 nm, 18 W) using PhotoRedOx Box EvoluChem (HepatoChem, Beverly, MA, USA) at room temperature. The extent of racemization was determined via HPLC using a CHIRAL ART Amylose-SA (φ: 4.6 × 250 mm, YMC) column and 100% CH_3_CN as the mobile phase [flow rate = 0.5 mL/min, respective retention times of 12.0 and 13.5 min for (−)-**1a** and (+)**-1a**].

### Isolation of **1b–d** as Enantiomers

**1b**: CHIRALPAK ID (1.0 × 50 cm), eluent: CH_3_CN, flow rate: 2.0 mL/min, temperature: 23 °C, detection: 254 nm, initial peak retention time = 23 min, and latter peak retention time = 29 min.

**1c**: CHIRALPAK IH (1.0 × 25 cm), eluent: CH_3_CN, flow rate: 2.0 mL/min, temperature: 23 °C, detection: 254 nm, initial peak retention time = 15 min, latter peak retention time = 22 min.

**1d**: CHIRALPAK IH (1.0 × 25 cm), eluent: CH_3_CN, flow rate: 2.0 mL/min, temperature: 23 °C, detection: 254 nm, initial peak retention time = 20 min, and latter peak retention time = 28 min.

### General Procedure for Enantiomeric Enrichment of **1b–d** Using the Recycle Photoreactor

Racemate **1b** (10 mg, 0.05 mmol) was injected into the recycling HPLC equipped with the photoreactor. The fractions of the desired enantiomer were accumulated after five cycles to yield (−)-**1b** as a colorless oil (9.1 mg, 91%, 98% ee).

(−)-**1c** was prepared according to the procedure described above for the preparation of (−)-**1b**. Using racemate **1c** as a colorless oil (10 mg, 0.06 mmol) yielded (−)-**1c** (8.5 mg, 85%, 98% ee) after five cycles.

(−)-**1d** was prepared according to the procedure described above for the preparation of (−)-**1b**. Using racemate **1d** as a colorless oil (10 mg, 0.06 mmol) yielded (−)-**1d** (7.9 mg, 79%, 99% ee) after six cycles.

(+)-**1c** was prepared according to the procedure described above for the preparation of (−)-**1b.** Using racemate **1c** (10 mg, 0.06 mmol) yielded (+)-**1c** (8.7 mg, 87 %, 99 % ee) after four cycles.

## Data Availability

The data from this study are available in the published article and its Supporting Information.

## Supporting Information Available

Determination of pseudo-first-order rate constants, *k*_obs_, examination of leaching of TPT^+^ from **A** to **C**, recycling of **C**, racemization in the glass tube containing various ratios of **C** and glass beads, enantiomeric enrichment of the sulfoxides in the recycle photoreactor, and description of the photoreactor (PDF)
