# Supplementary material for: Conversion of Racemic Alkyl Aryl Sulfoxides into Pure Enantiomers Using a Recycle Photoreactor: Tandem Use of Chromatography on Chiral Support and Photoracemization on Solid Support
Source: J Org Chem. 2023 May 8;88(11):6955–61. doi: 10.1021/acs.joc.3c00265 (PMC10242752; doi:10.1021/acs.joc.3c00265)
Supplement: Supplementary file 1 — jo3c00265_si_001.pdf [file jo3c00265_si_001.pdf]

Supporting Information

Conversion of racemic alkyl aryl sulfoxides to pure enantiomers using a recycle photoreactor: Tandem use of chromatography on chiral support and photoracemization on solid support.

Kumi Tozawa,[1] Kosho Makino,[2] Yuki Tanaka,[1] Kayo Nakamura,[1] Akiko Inagaki,[3] Hidetsugu Tabata,[4] Tetsuta Oshitari,[4] Hideaki Natsugari,[5] Noritaka Kuroda,[6] Kunio Kanemaru,[7] Yuji Oda,[7] and Hideyo Takahashi*[1]

[1]Faculty of Pharmaceutical Sciences, Tokyo University of Science, 2641Yamazaki, Noda-shi, Chiba 278-8510, Japan

[2]Research Institute of Pharmaceutical Sciences, Musashino University, Nishitokyo, Tokyo 202-8585, Japan

[3]Faculty of Science and Technology, Seikei University, 3-3-1 Kichijoji Kitamachi, Musashino-shi, Tokyo 180-8633, Japan

[4]Faculty of Pharma Sciences, Teikyo University, 2-11-1 Kaga, Itabashi-ku, Tokyo 173-8605, Japan

[5]Graduate School of Pharmaceutical Science, The University of Tokyo, 7-3-1 Hongo, Bunkyo-ku, Tokyo 113-0033, Japan

[6]YMC Co., Ltd., 284 Daigo, Karasuma Nishiiru Gojo-dori, Shimogyo-ku, Kyoto 600-8106, Japan

[7]IWASAKI ELECTRIC CO., LTD.,1-1, Ichiriyama-cho, Gyoda-shi, Saitama, 361-8505, Japan

Corresponding Author

E-mail: hide-tak@rs.tus.ac.jp

Contents

1. **Determination of pseudo-first-order rate constants, k_{obs}……………………..……...S3**
2. **Examination of leaching of TPT$^+$ from immobilized catalysts A~C…….….…..S6**
3. **Recycle of catalysts C…………………………………………………………….S9**
4. **Racemization through glass tube containing various ratio of catalyst C and glass bead. (Table 2)……………..........…………………...………………..…..................S10**
5. **Enantiomeric enrichment of sulfoxide in recycle photoreactor（Table 3）….S13**
6. **Account of photoreactor………………………………………………………....S17**

1. Determination of pseudo-first-order rate constants, k_{obs}.

Racemization rate (k_{obs}) of (+)-**1a** (0.01 M) with catalyst **A** at r.t. in MeCN.

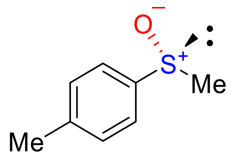

(+)-**1a**

First-order dependence of the rate of racemization on catalyst **A** for the reaction of (+)-**1a**

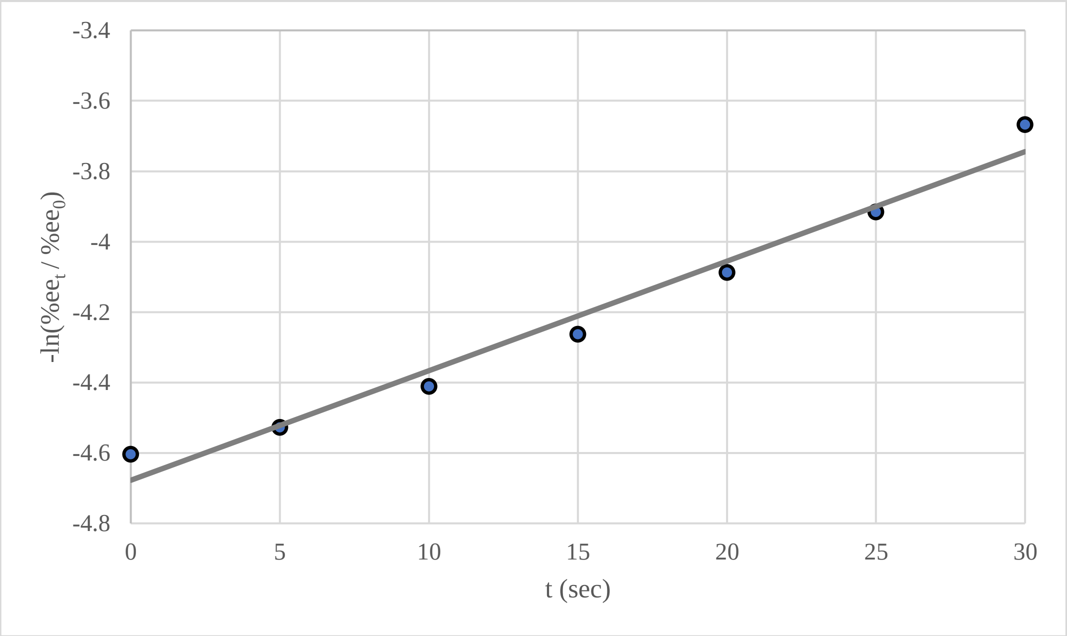

Figure S1

Racemization rates were shown to follow pseudo-first-order rate constants, k_{obs} were determined from the slope of the straight line obtained when -ln(%ee_t/%ee_0) is plotted against time.

$k_{obs} = 2.59 \times 10^{-2}$ (s^{-1})

Racemization rate (k_{obs}) of (+)-**1a** (0.01 M) with catalyst **B** at r.t. in MeCN.

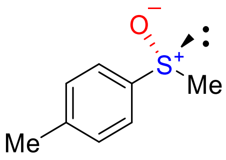

(+)-**1a**

First-order dependence of the rate of racemization on catalyst B for the reaction of (+)-**1a**

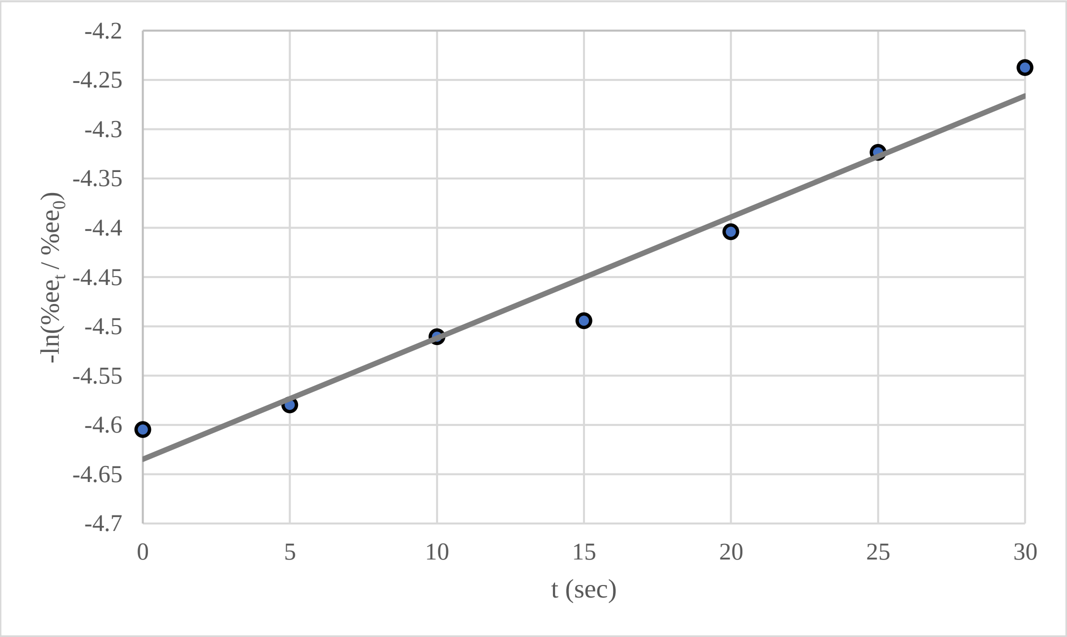

Figure S2

Racemization rates were shown to follow pseudo-first-order rate constants, k_{obs} were determined from the slope of the straight line obtained when $-\ln(\%ee_t/\%ee_0)$ is plotted against time.

$k_{obs} = 9.73 \times 10^{-3}$ (s^{-1})

Racemization rate (k_{obs}) of (+)-**1a** (0.01 M) with catalyst **C** at r.t. in MeCN.

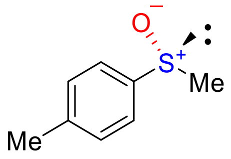

(+)-**1a**

First-order dependence of the rate of racemization on catalyst C for the reaction of (+)-**1a**

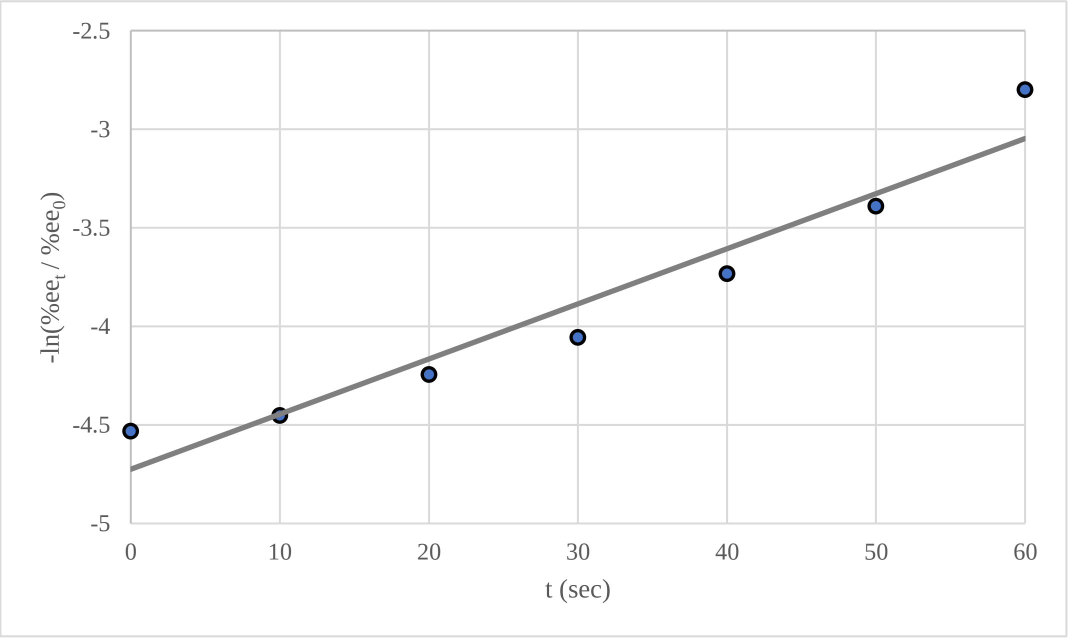

Figure S3

Racemization rates were shown to follow pseudo-first-order rate constants, k_{obs} were determined from the slope of the straight line obtained when -ln(%ee$_t$/%ee$_0$) is plotted against time.

$k_{obs=}$ 1.99 × 10^{-2} (s^{-1})

2. Examination of leaching of TP+ from immobilized catalysts A~C.

<Immobilized catalyst A>

Preparation of Filtrate **A'** from immobilized catalyst **A**, and examination of its catalytic activity (general procedure).

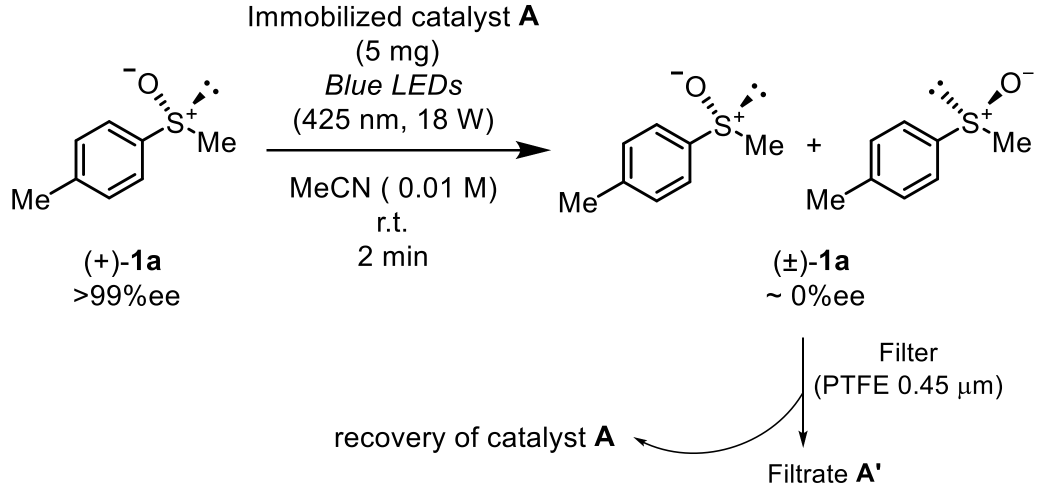

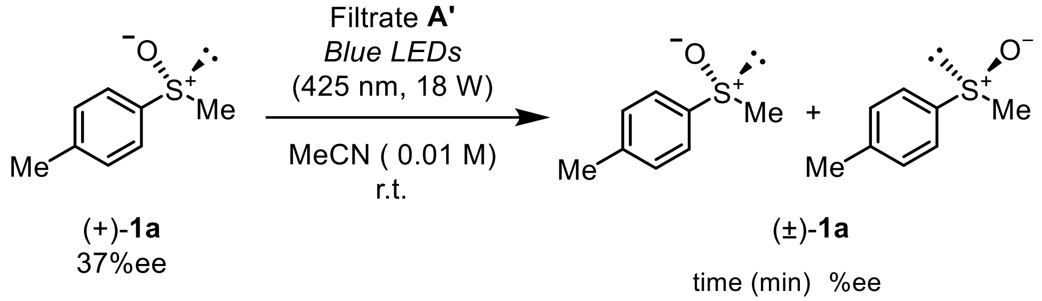

time (min)	%ee
0	37
2	3
30	2

37%ee of (+)-**1a** was dissolved in the filtrate A' (CH_3CN solution) and was irradiated (425 nm). A change of %ee of (+)-**1a** is shown below.

Figure S4

Chiral HPLC charts of (+)-**1a** by 0 min, 2 min, 30 min.

0 min 2 min 30 min

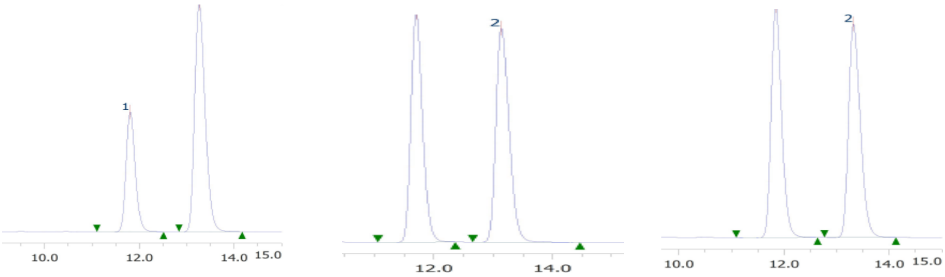

31.5 : 68.5 (37 %ee) 48.5 : 51.5 (3 %ee) 48.9 : 51.1 (2 %ee)

CHIRAL ART Amylose-SA (ϕ 4.6 mm × 250 mm)

Eluent : MeCN

Flow rate : 0.5 ml/min. Rt:12.0 min for (-)-**1a**, 13.5 min for (+)-**1a**

Figure S5

<Immobilized catalyst B>

According to the general procedure mentioned above, leaching of TPT^+ from immobilized catalyst **B** was examined.

40%ee of (+)-**1a** was dissolved in the filtrate B' (CH_3CN solution) and was irradiated (425 nm). A change of %ee of (+)-**1a** is shown below.

Chiral HPLC charts of (+)-**1a** by 0 min, 2 min, 30 min.

0 min 2 min 30 min

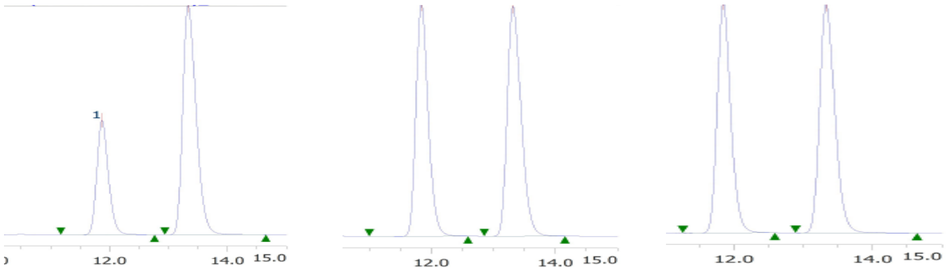

30.1:69.9 (40 %ee) 47.0:53.0 (6 %ee) 47.2:52.8 (6 %ee)

CHIRAL ART Amylose-SA (ϕ 4.6 mm × 250 mm)

Eluent : MeCN

Flow rate : 0.5 ml/min. Rt: 12.0 min for (-)-**1a**, 13.5 min for (+)-**1a**

Figure S6

<Immobilized catalyst C>

According to the general procedure mentioned above, leaching of TPT^+ from immobilized catalyst **C** was examined.

70%ee of (+)-**1a** was dissolved in the filtrate C' (CH_3CN solution) and was irradiated (425 nm). A change of %ee of (+)-**1a** is shown below.

Chiral HPLC charts of (+)-**1a** by 0 min, 2 min, 30 min.

0 min 2 min 30 min

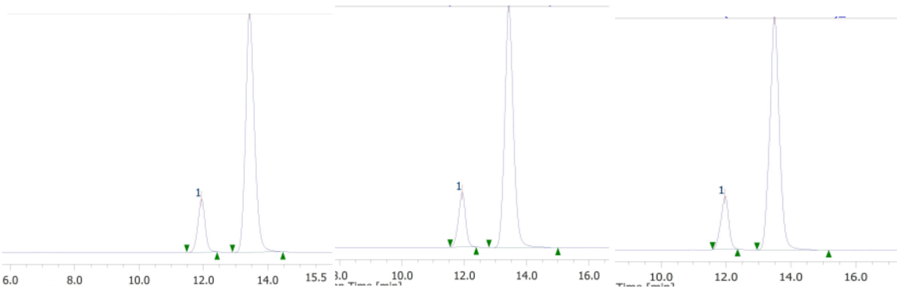

15.3:84.7 (70 %ee) 15.7:84.3 (69 %ee) 15.6:84.4 (69 %ee)

CHIRAL ART Amylose-SA (ϕ 4.6 mm × 250 mm)

Eluent : MeCN

Flow rate : 0.5 ml/min. Rt: 12.0 min for (-)-**1a**, 13.5 min for (+)-**1a**

Figure S7

3. **Recycle of catalysts C.**

Table S1

Racemization of **1a** by recycled immobilized catalyst **C**

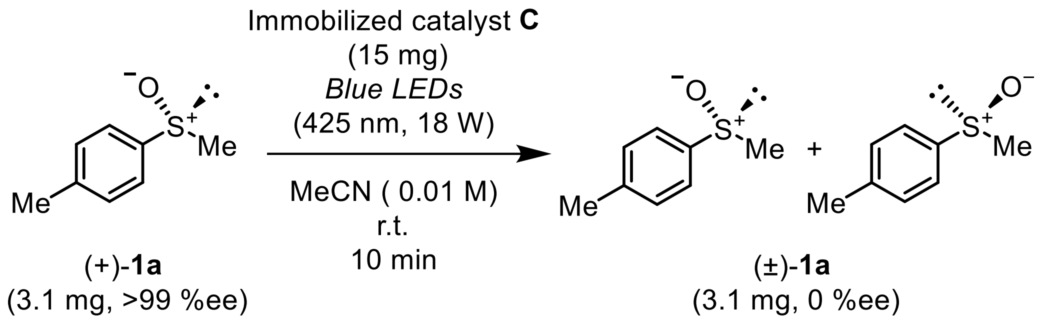

CHIRALPAK IG (ϕ 4.6 mm × 250 mm)

Eluent: MeCN   Flow rate : 0.5 ml/min. Rt: 16.2 min for (+)-**1a**, 19.0 min for (-)-**1a**

Number of use	1	2	3	4	5	6	7	8	9	10
%ee	+0.2	-0.2	0.0	0.0	+0.2	+0.2	0.0	0.0	-0.2	-0.2

4. Racemization through glass tube containing various ratio of catalyst C and glass bead. (Table 2)

HPLC chart of **1b** (Entry 1)

CHIRALPAK ID (ϕ 4.6 mm × 500 mm)

Eluent: MeCN   Flow rate : 2.0 ml/min. Rt: 52 min for (+)-**1b**, 58 min for (-)-**1b**

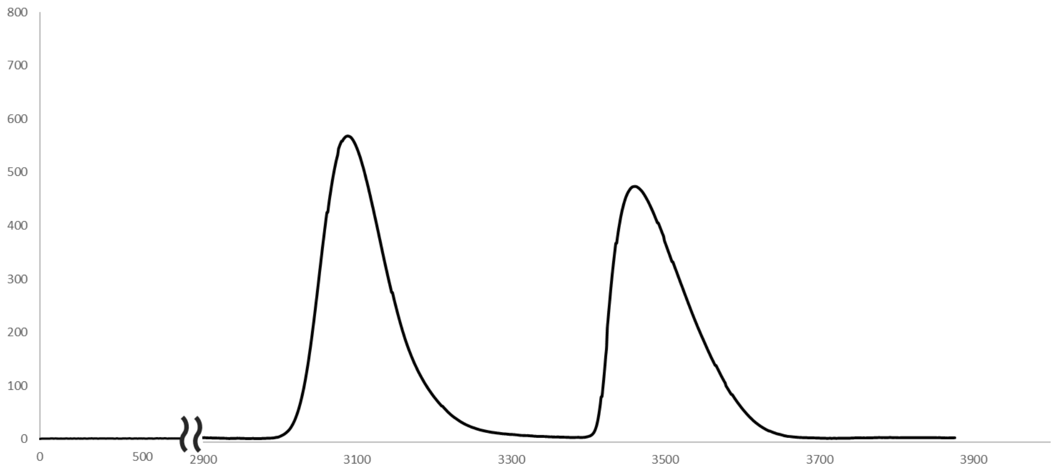

53 : 47 (6 %ee)

Figure S8

HPLC chart of **1b** (Entry 2)

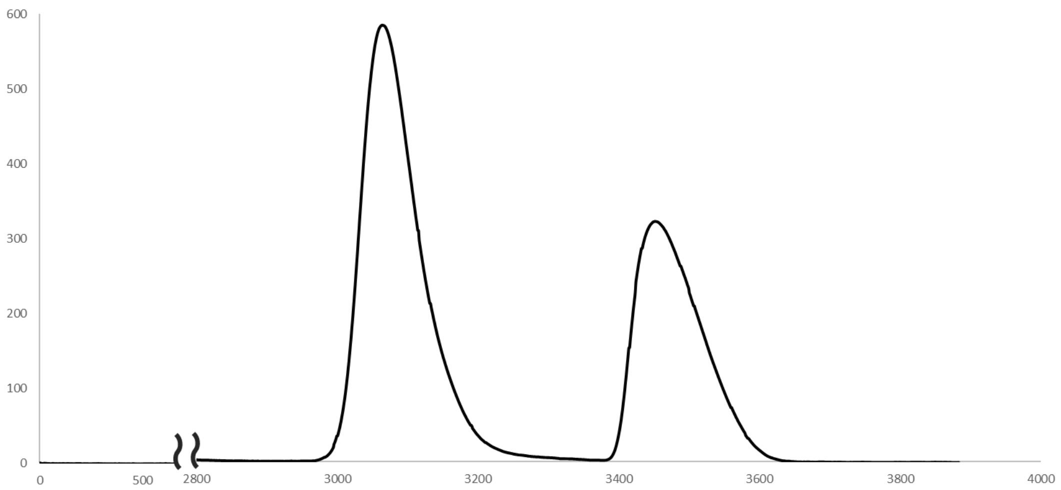

62 : 38 (24 %ee)

Figure S9

HPLC chart of **1b** (Entry 3)

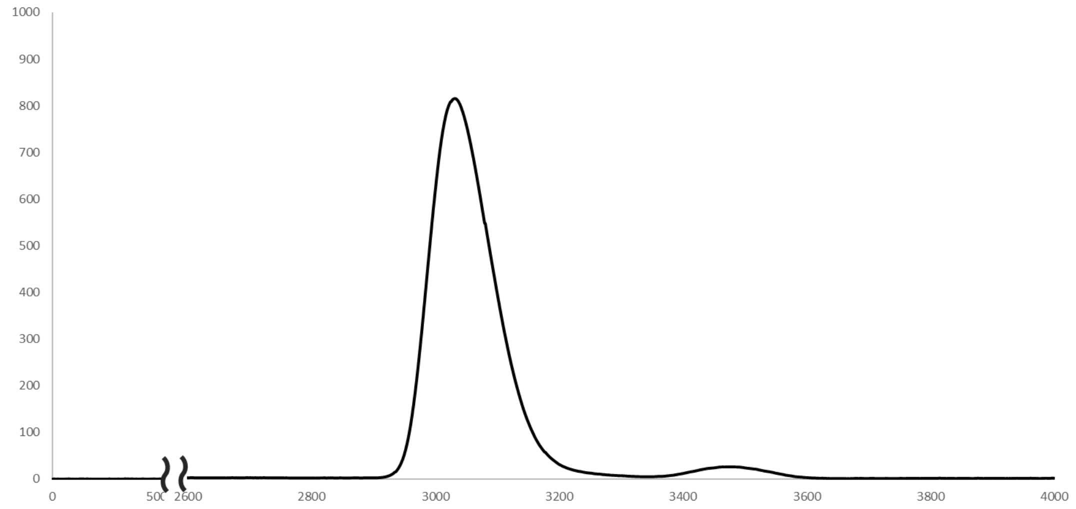

96 : 4 (92 %ee)

Figure S10

HPLC chart of **1c** (Entry 4)

CHIRALPAK IH (ϕ 4.6 mm × 250 mm)

Eluent: MeCN   Flow rate : 2.0 ml/min. Rt: 36 min for (+)-**1c**, 43 min for (-)-**1c**

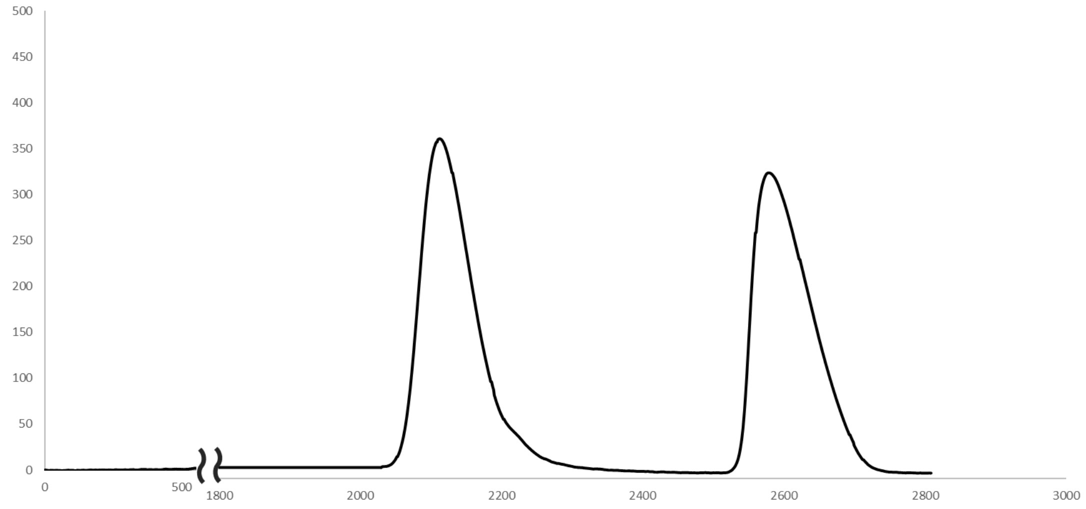

52 : 48 (4 %ee)

Figure S11

HPLC chart of **1d** (Entry 5)

CHIRALPAK IH (ϕ 4.6 mm × 250 mm)

Eluent: MeCN   Flow rate : 2.0 ml/min. Rt: 45 min for (+)-**1d**, 52 min for (-)-**1d**

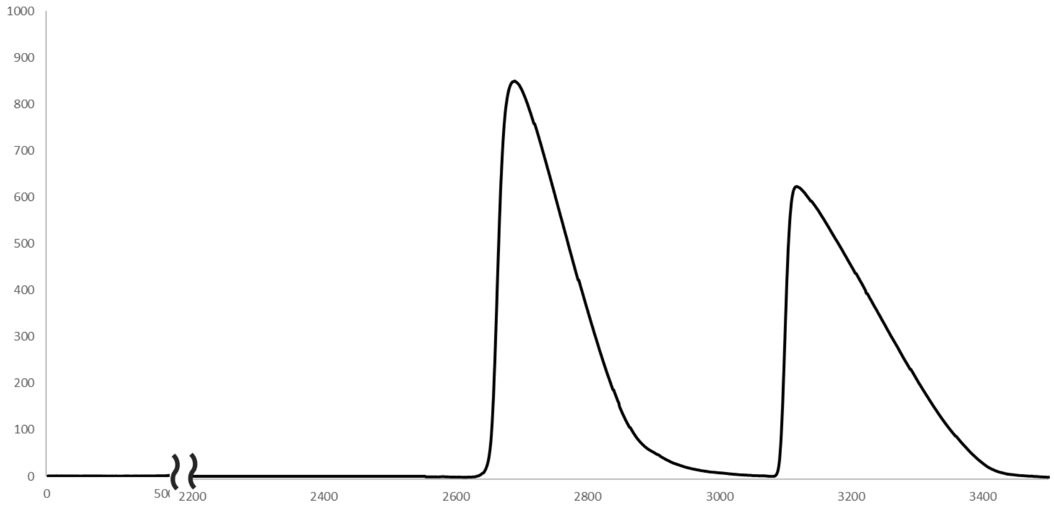

53 : 47 (6 %ee)

Figure S12

5. Enantiomeric enrichment of sulfoxide in recycle photoreactor. （Table 3）

(-)-**1b**

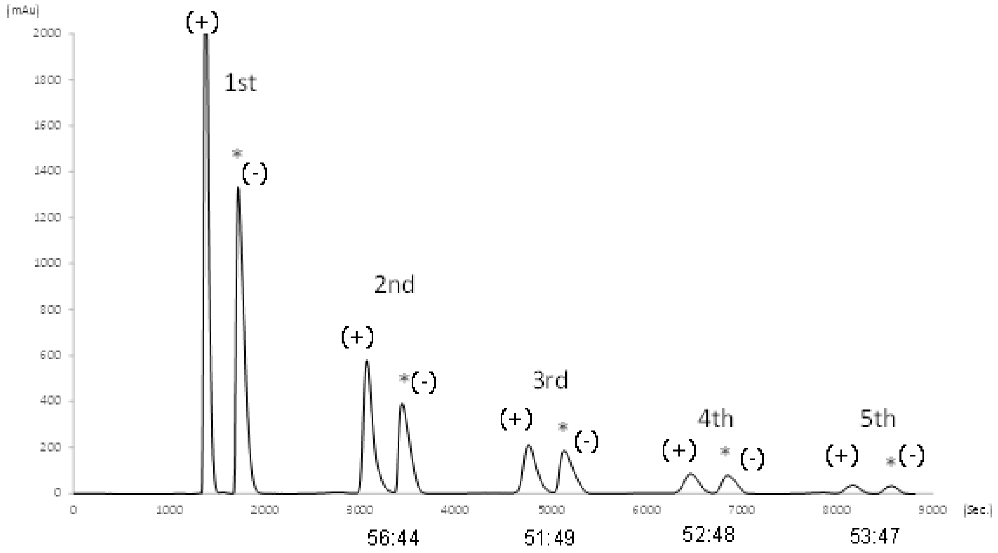

Column: CHIRALPAK ID (ϕ 10 mm, length 50 cm),

Eluent: CH_3CN,

Flow rate: 2.0 ml/min.

fraction denoted by an asterisk is accumulated.

Figure S13

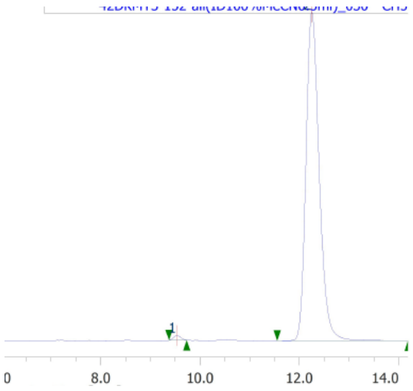

Column: CHIRALPAK ID (ϕ 5 mm, length 25 cm),

Eluent: CH_3CN,

Flow rate: 2.0 ml/min.

Rt: 9.5 min for (+)-**1b**, 12.2 min for (-)-**1b**

0.8 : 99.2 (98.4 %ee)

Figure S14

(-)-1c

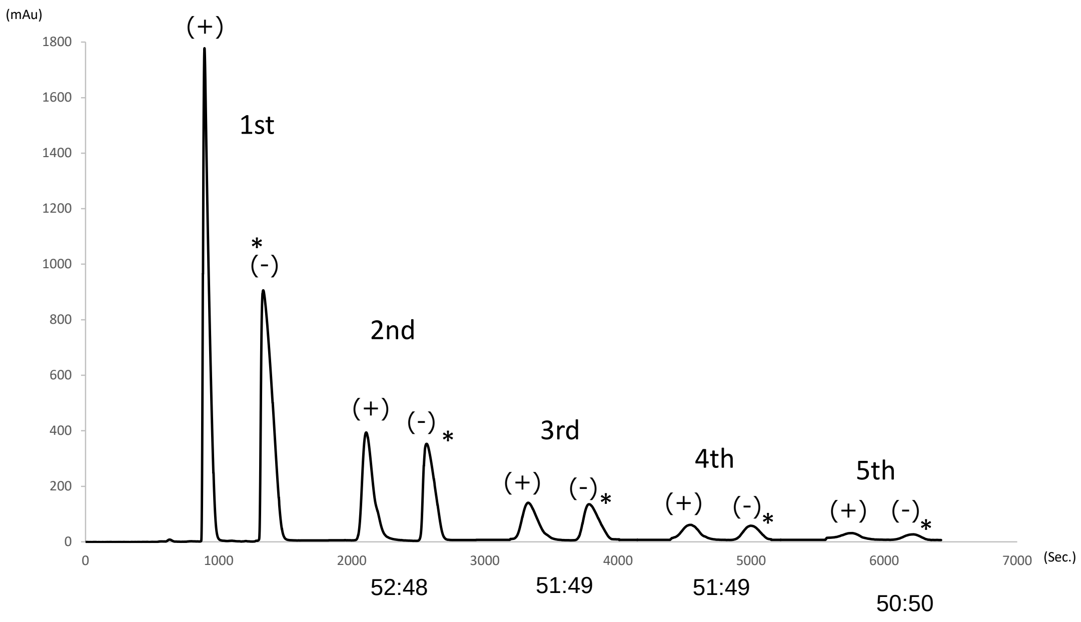

Column: CHIRALPAK IH (ϕ 10 mm, length 25 cm),

Eluent: CH_3CN,

Flow rate: 2.0 ml/min.

fraction denoted by an asterisk is accumulated.

Figure S15

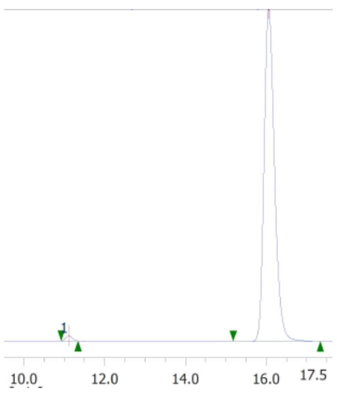

Column: CHIRALPAK IH (ϕ 5 mm, length 25 cm),

Eluent: CH_3CN,

Flow rate: 2.0 ml/min.

Rt: 11.1 min for (+)-**1c**, 16.1 min for (-)-**1c**

1.0 : 99.0 (98.0 %ee)

Figure S16

(-)-1d

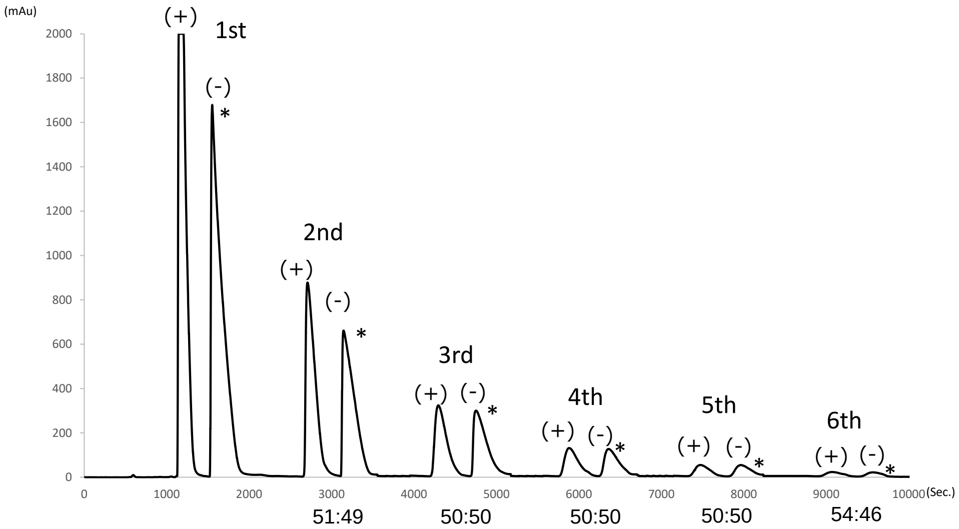

Column: CHIRALPAK IH (ϕ 10 mm, length 25 cm),

Eluent: CH_3CN,

Flow rate: 2.0 ml/min.

fraction denoted by an asterisk is accumulated.

Figure S17

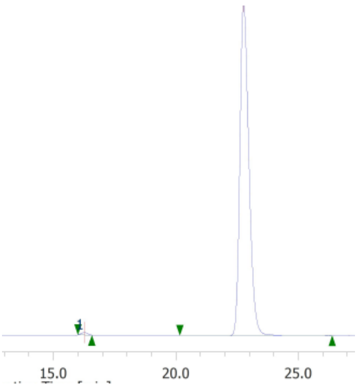

Column: CHIRALPAK IH (ϕ 5 mm, length 25 cm),

Eluent: CH_3CN,

Flow rate: 2.0 ml/min.

Rt: 16.3 min for (+)-**1d**, 22.8 min for (-)-**1d**

0.6 : 99.4 (98.8 %ee)

Figure S18

(+)-1c

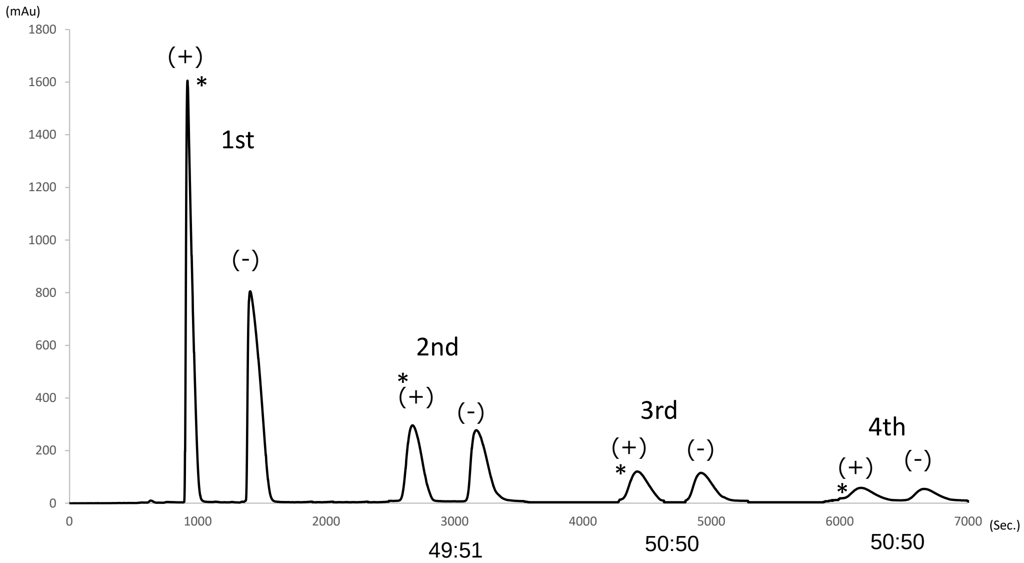

Column: CHIRALPAK IH (ϕ 10 mm, length 25 cm),

Eluent: CH_3CN,

Flow rate: 2.0 ml/min.

fraction denoted by an asterisk is accumulated.

Figure S19

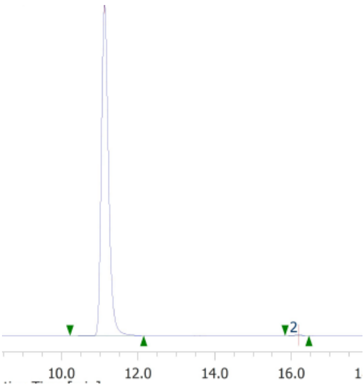

Column: CHIRALPAK IH (ϕ 5 mm, length 25 cm),

Eluent: CH_3CN,

Flow rate: 2.0 ml/min.

Rt: 11.1 min for (+)-**1c**, 16.2 min for (-)-**1c**

99.8 : 0.2 (99.6 %ee)

Figure S20

6. Account of photoreactor

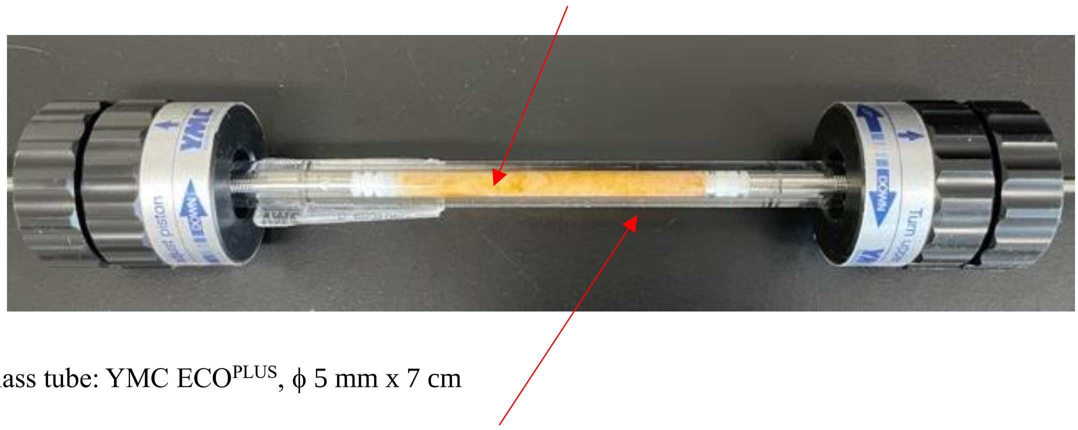

Glass tube: YMC ECO^{PLUS}, ϕ 5 mm x 7 cm

Figure S21

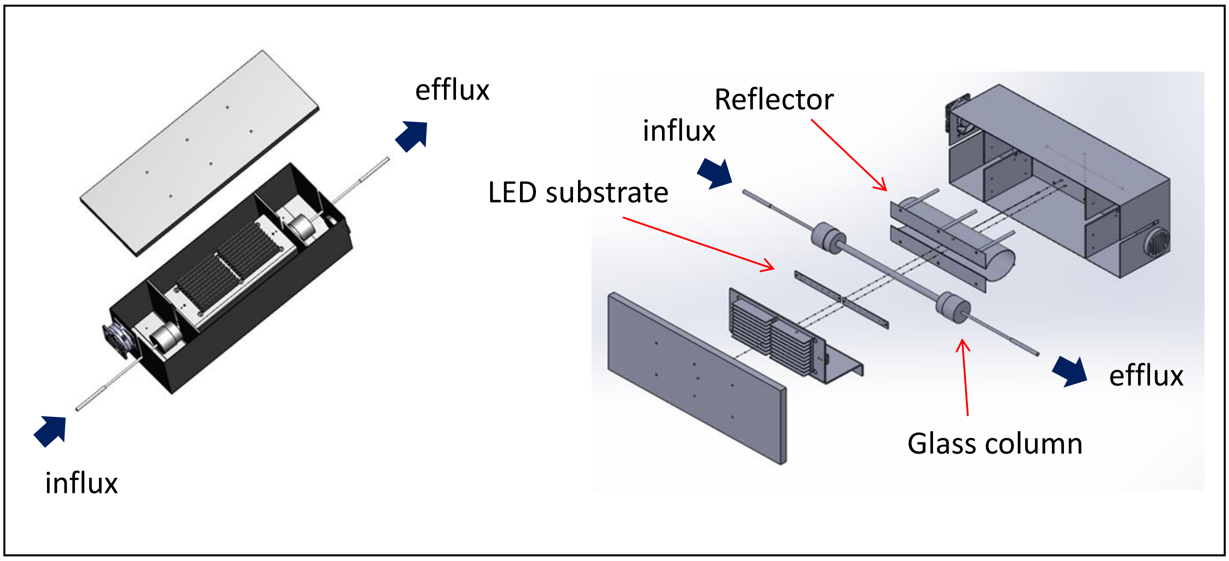

Figure S22
